# From words to sentences: designing and evaluating a parent-facing sign language learning app

**DOI:** 10.1007/s10209-026-01378-9

**Published:** 2026-08-21

**Authors:** Jos Ritmeester, Beyza Sümer, Marije Boonstra, Maartje de Meulder, Belinda van der Aa, Floris Roelofsen

**Affiliations:** 1 University of Amsterdam, Amsterdam, The Netherlands; 2 Royal Auris Group, Rotterdam, The Netherlands; 3 HU University of Applied Sciences Utrecht, Utrecht, The Netherlands

**Keywords:** Sign language learning, Hearing parents of deaf children, Mobile applications, Mobile-assisted language learning, App design

## Abstract

To prevent language deprivation, hearing parents of deaf and hard of hearing (DHH) children need to learn sign language as soon as possible after their child is identified as DHH. However, sign language resources for parents are limited and often lack focus on sentence structure. To address these gaps, we have been developing ZINinNGT, a mobile Dutch Sign Language (NGT) learning app designed specifically for parents and focused on sentence formation. In this paper, we describe the app’s initial design principles, report on feedback from hearing parents and NGT teachers on the ZINinNGT prototype, and present an updated set of design principles based on this feedback. Twenty-one hearing parents of DHH children and six NGT teachers participated in our study and individually tried the prototype while verbalizing their thoughts and actions. Participants responded positively to the app’s sentence-based approach and appreciated features that accommodated different learning preferences, such as the adjustable playback speed and camera angle. Moreover, they appreciated that the content was applicable to the daily lives of parents with young children. At the same time, they expressed a desire for a more holistic, visual and child-friendly app. They wished for an app that supported both vocabulary learning and sentence construction, included little text, and could be used together with their child. Based on their feedback, we propose eight design principles for parent-facing sign language learning tools. Our findings suggest that a sentence-focused app can be a valuable complement to courses, addressing the need for more parent-oriented resources that go beyond vocabulary learning.

## Introduction

The significance of early language development is well described in the child language literature. Research indicates that while early language skills are important in their own right, they also support a child’s social-emotional and cognitive development, and predict future language development [1–3]. Deaf and hard of hearing (DHH) children who receive early sign language input follow the same language milestones as hearing children acquiring spoken language in the same order and at a comparable rate [4–6]. However, 90–95% of DHH children are born to hearing parents [7], who generally do not know a sign language. In fact, for many of these parents, the birth of their DHH child is the first experience they have with sign language and the Deaf community [8]. As a result, DHH children often experience a delay in acquiring their first language, which can have life-long consequences for the child’s linguistic, social-emotional and cognitive development [1, 2, 9–11].

To prevent language deprivation, it is crucial that hearing parents of DHH children learn a sign language as early as possible. Yet, learning a sign language presents many challenges for parents. Relatively few courses and resources exist, and those that are available are generally designed for adult learners in general rather than specifically for parents [12]. This distinction matters: while other learners typically focus on communication with adults, parents need resources that prepare them for interaction with a young child, requiring different vocabulary, sentence constructions, and communicative situations.

Moreover, existing sign language materials (such as books, YouTube videos, and apps) are often overly focused on vocabulary learning rather than on sentence formation. Parents often rely on web-based dictionaries for sign language learning [13], and even during formal, offline courses, the lack of instruction on language structure is a common frustration among parents [14–16]. Similarly, Oyserman and de Geus [17] found that while Dutch parents had learned various signs during their Dutch Sign Language (*Nederlandse Gebarentaal*, NGT) courses, they barely learned the syntactical, semantic, and morphological features of NGT.

Further focusing on the Dutch context, in a previous publication [16], we examined hearing parents’ experiences learning NGT through semi-structured interviews with 21 parents and six NGT teachers. Parents reported that existing NGT courses offered too little—and too late—grammar instruction, that courses often misaligned with their children’s age and that course materials were often outdated. When discussing self-study resources, parents valued materials that were fun and could be used together with their child. However, they also noted that resources were often difficult to access (e.g., requiring lengthy login procedures or being restricted to computer use rather than mobile devices) and limited in scope. NGT teachers largely echoed these concerns, pointing to an overemphasis on vocabulary, outdated materials, and a lack of content specifically designed for parents.

Together, this context underscores the need for up-to-date tools that are easily accessible, parent-focused, and supportive of sentence-level learning. Therefore, we have developed *ZINinNGT*,^1^ an NGT learning app specifically designed for hearing parents of DHH children. The app is intended to complement offline NGT courses for parents of DHH children aged one to five years. By providing example sentences containing core vocabulary and grammatical constructions, ZINinNGT aims to strengthen parents’ practical NGT skills and thereby indirectly support DHH children’s access to NGT. Furthermore, because all sentences are typical of parent–child conversations and thus directly applicable in discourse, ZINinNGT seeks to contribute to the improvement of parent–child interaction.

Within this paper, we describe the development and evaluation of the ZINinNGT prototype. The key contributions of this paper are the following:

1. We present and evaluate a set of interaction techniques designed to support parents in learning full sentences in NGT. These include options such as viewing from multiple camera angles, adjusting playback speed, toggling between sentence-level and sign-for-sign subtitles, and searching by grammatical features.
2. We distill eight design principles for parent-facing sign language learning tools: grammar-aware input, perceptual control (adjustable camera angles, playback speed, subtitle options), need-driven retrieval (find what you need, when you need it), holistic design (integrating sentences, lexicon, and practice components), design for learner motivation (implementing gamification elements), visual-first (low text), variant-aware (supporting regional and standard variants), and joint parent–child use.
3. We provide empirical evidence from a user study with parents and teachers, linking specific breakdowns (e.g., text-heavy interface, not child-friendly) to concrete UI changes and improved task performance.

The paper is structured as follows. We begin with describing related work in the next section. Section 3 presents the initial design objectives of ZINinNGT and describes the design of the prototype. Section 4 outlines the methodology of our user study, and Sect. 5 presents the results. Finally, Sects. 6 and 7 provide the discussion and conclusion.

## Related work

### Existing sign language learning apps for parents

In the Netherlands, two popular sign language learning apps have been developed specifically for DHH children,^2^ and these are also frequently used by hearing parents—often as a shared activity with their children—to learn child-related NGT vocabulary [16]. Both apps focus on vocabulary learning through play and are designed to appeal to young children by incorporating games, engaging images and colors, and, in the case of the *KinderGebaren* app, videos of children demonstrating the signs [18, 19].

Outside of the Netherlands, several initiatives have focused on developing sign language learning apps. Here, we describe some that are particularly relevant to ZINinNGT because they are either intended for hearing parents of DHH children or because they extend beyond the vocabulary level by providing sentences and grammar explanations. Examples of sign language learning apps aimed at hearing parents include *SMARTSign* and *PopSign* for ASL, and *NextSense Auslan Tutor* for Australian Sign Language (Auslan) [20–22]. *Auslan Tutor* is designed for parents learning Auslan and focuses on vocabulary learning, with key signs divided into semantic themes. It includes features such as quizzes, adjustable playback speed, and multiple camera angles that allow users to slide around the signer. It also provides linguistic information such as handshape information, sign variants, and example sentences showing sign use.

*PopSign* is a bubble-shooter game aimed at hearing parents of DHH children that draws on vocabulary from the MacArthur-Bates Communicative Development Inventories [23], a set of vocabulary inventories that includes concepts typically acquired early in children’s language development. *PopSign* supports both receptive and expressive ASL vocabulary development by making use of sign recognition [24]. *SMARTSign* is another vocabulary-focused ASL app designed to help hearing parents of DHH children learn and practice signs. It features a search function, quizzes, and a practice component that allows parents to record and watch videos of themselves signing. Future development plans include expanding the app to incorporate ASL grammar as well [20].

Several commercial sign language apps have also been developed, though these are often aimed at a general audience rather than specifically at hearing parents. *Toleio* is a gamified app for Norwegian Sign Language that includes a dictionary, quizzes, simple sentences and dialogues, as well as modules on grammar and Deaf^3^ culture and history [25]. Although *Toleio* does mention ‘parents and children’ among their target groups, its content is designed for a more general audience, as it does not seem to include content specifically tailored to hearing parents of DHH children. The company behind *Toleio*, SignLab, also created very similar apps for other sign languages, including *ASL Bloom* for ASL, *yoDGS* for German Sign Language, and *ISL Journey* for Indian Sign Language [26].

*Lingvano* supports ASL, British Sign Language, and Austrian Sign Language [27]. It is also highly gamified, featuring quizzes, rewards, and the possibility to set learning goals. In addition, it offers dictionary searches, example sentences and conversations, and grammar explanations. Lastly, *PocketSign* is a gamified app for ASL that includes lessons on vocabulary, simple phrases, and ASL grammar. It also tracks users’ learning progress, gives rewards, and features a dictionary and quizzes, including both sign comprehension and sign production quizzes that provide feedback based on sign recognition [28].

In sum, both within the Netherlands and internationally, a range of promising initiatives have been developed to support hearing parents of DHH children in learning sign language. These tools often incorporate engaging and innovative features (such as games, quizzes, multiple camera angles, and sign recognition) which make them attractive and accessible for families. However, despite their strengths, most existing apps remain limited to vocabulary learning, offering little support for developing sentence-level skills. Some apps do address sentence formation, but they target a broad audience and are not tailored to child-focused communication. This highlights the need for next-generation tools that specifically support parents and go beyond individual signs to foster broader communicative competence in sign language. Table 1 provides an overview of the apps and their features mentioned in this subsection, highlighting parent-focused content and support beyond vocabulary learning.

**Table 1:** Summary of existing tools and features

	Tailored to parents/children	Beyond vocabulary
Lotte en Max Kindergebaren app	✓	✘
KinderGebaren-app	✓	✘
Auslan Tutor	✓	✓^a^
PopSign	✓	✘
SMARTSign	✓	✘
Toleio	✘	✓
Lingvano	✘	✓
PocketSign	✘	✓

^a^This app is vocabulary-focused but provides simple example sentences

### Design principles for language learning apps

Jamaldeen et al. [29] conducted a study similar to the present study, in which they present a set of guidelines for designing mobile language learning applications. These guidelines are derived from principles identified in the literature and expanded with insights from their evaluation study of an English learning app they developed. The authors distinguish between technological, pedagogical, and human-centered facets of mobile learning design. For the purposes of this paper, we focus on the pedagogical and human-centered facets they identified, as these are most relevant to our work.

The pedagogical principles identified in their literature review include ‘keeping information organized’, which involves presenting content in a structured way and chunking and grouping of information, and ‘multiple pedagogy/activities’, which refers to accommodating different learning styles and incorporating diverse exercises to keep the learning interactive. Interactivity is an important feature of language learning apps, as it helps sustain learner engagement and supports learning retention [30].

For the human-centered facets, the authors in [29] highlight the design principles of ‘design for personalized learning’ and ‘incorporate collaborative activities’. Personalization entails tailoring the learning experience to the unique needs and preferences of individual learners [30]. According to the authors, this can, for example, be achieved by adding a bookmark function to allow users to organize lessons according to their preferences. Other approaches to personalization include the use of learning algorithms, which automatically adapt the difficulty of learning exercises based on users’ performance, or the adaptation of content based on users’ areas of interest (which users are sometimes asked about when creating an account) [30]. Collaborative activities can, for example, be supported through the integration of social media-based platforms and online forums. Through these platforms, learners can interact with one another as well as with teachers or native users of the target language [30].

Based on findings from their own evaluation study, the authors in [29] additionally propose ‘design for learner motivation’ as a human-centered design principle, which can be supported through game-based learning and by providing users with information about their learning progress. Several studies on mobile assisted-language learning applications also emphasize the importance of including gamification—implementing game elements in non-game contexts—to increase learning motivation and commitment [30, 31]. Examples of gamification elements that have been found to be positively perceived include rewards, badges, learning streaks, and leader boards, which create a sense of competition [31]. Lastly, the authors also add ‘design for supporting medium of learning’ to the pedagogical principles, concluding that mobile learning apps are most beneficial when used within a blended learning environment (combining mobile and e-learning with face-to-face courses) rather than as a primary means of learning.

To summarize, Table 2 presents the design principles described in this subsection.

**Table 2:** Summary of design principles identified by Jamaldeen, Hewagamage, and Ekanayaka [29]; table adapted from the original publication

Category	Design principle
Pedagogical	Keep information organized
	Multiple pedagogies/activities
	Design for supporting medium of learning
Human-centered	Design for personalized learning
	Incorporate collaborative activities
	Design for learner motivation

## ZINinNGT prototype description

In this section, we describe our initial design objectives and provide an overview of the prototype’s design and main features. Finally, we map the design objectives onto the prototype’s features to illustrate how the objectives are operationalized.

### Design objectives

Based on the literature discussed in Sects. 1 and 2, we identify several design objectives for our parent-facing app.

First, because parents and NGT teachers express the need for sign language materials that move beyond isolated vocabulary [14–16], the app should do so and provide **grammar-aware input**. This includes NGT sentences ranging from simple (suitable for beginners and parents of very young children) to more complex sentences (for advanced learners and parents of slightly older children). In addition, grammar explanation videos should be included to help parents understand the key grammar constructions in these sentences, as well as to reinforce explanations from their sign language courses.

Second, to accommodate different learning styles and preferences—identified by Jamaldeen, Hewagamage, and Ekanayaka [29] as core design principles—the app should support **perceptual control**. Features include multiple camera angles, adjustable playback speed, and optional subtitles (at sentence- or word-level).

Third, the app should enable **need-driven retrieval**. Parents should be able to find what they need when they need it. This design principle aligns with findings reported in an earlier publication, in which parents reported that they valued resources that are quick and easy to access [16]. To meet this need, the app must offer a well-organized interface, an intuitive search function, and a sufficiently broad scope so that parents can easily find sentences relevant to their immediate communicative needs. In line with the personalized-learning principle described by Jamaldeen, Hewagamage, and Ekanayaka [29], the app should also support bookmarking, enabling parents to organize and revisit content based on their individual needs.

Finally, although the literature emphasizes the value of interactive activities and motivational elements [29–31] we chose not to incorporate quizzes or motivational features in this prototype. Hearing parents of DHH children are often highly motivated to learn sign language, drawing their primary reward from successful communication with their child rather than from gamified features. For this reason, we initially prioritized the other design objectives. As shown in the Results and Discussion (Sects. 5 and 6), we later reconsidered this decision.

Although not a design objective, it is important to briefly address the app’s content. Because ZINinNGT targets parents of young children, all app content focuses on parent–child interactions and their daily routines. Zarchy and Geer [11] formulated guidelines for family-centered sign language curricula, in which they repeatedly emphasize the importance of content centered around common daily routines. This ensures that the learning material can be integrated immediately and repeatedly into parents’ daily lives, making language learning most efficient.

### Prototype design

The ZINinNGT prototype was created in collaboration with YipYip, a Dutch company specializing in app development. ZINinNGT is suitable for both Android and iOS smartphones. In this section, we highlight the prototype’s main features.

#### Search bar and filters

**Fig. 1:**
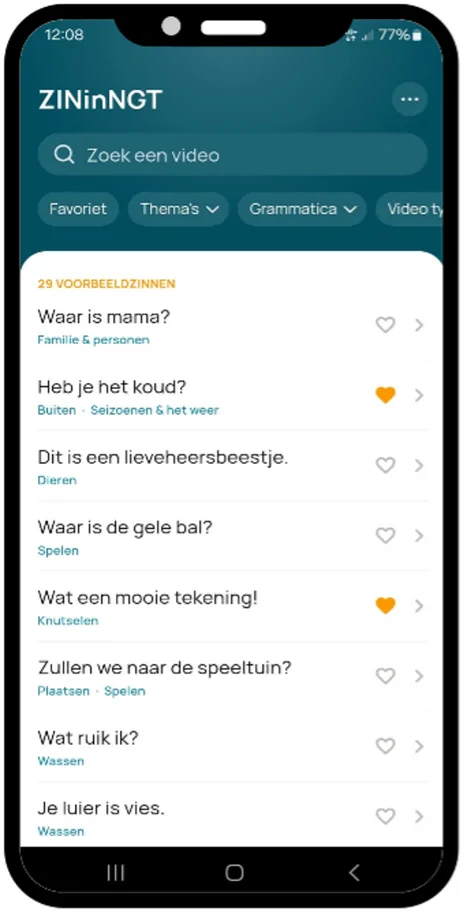
Home screen of ZINinNGT

The app features a search function, as shown in Fig. 1, which allows users to search for two types of videos: (1) videos of example sentences in NGT and (2) videos explaining NGT grammar concepts.

**Fig. 2:**
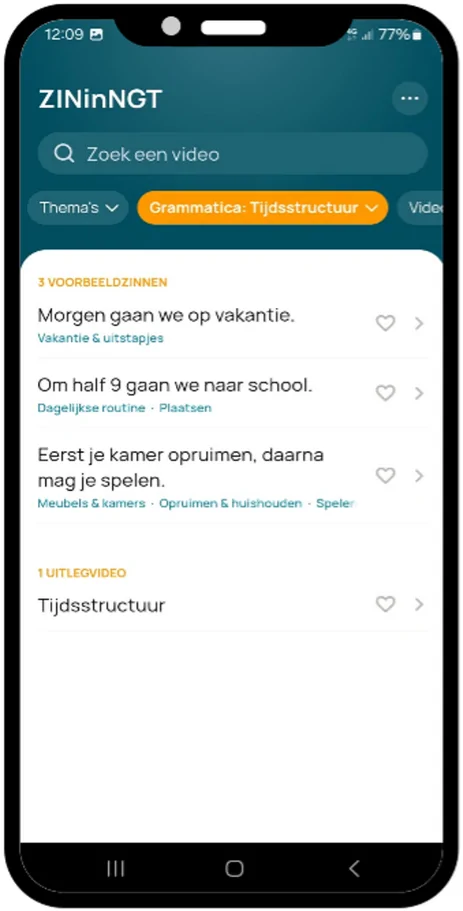
Example use of grammar filter

Users can search for videos using the search bar, by selecting search filters, or by combining a search term with filters. The prototype offers different search filters, such as “Favorite”, “Themes” and “Grammar”. The “Favorite” filter allows users to only view videos they have marked as favorites, serving as a bookmark function. The “Themes” filter allows users to search for sentences relevant to a specific semantic theme. Through the “Grammar” filter, users can search for example sentences and grammar explanation videos based on specific grammatical components. For instance, selecting "Time expressions" will retrieve all example sentences including time expressions as well as related grammar explanation videos (as shown in Fig. 2).

The main idea behind the search bar and the “Grammar” filter is to help users find signs and grammar concepts learned in NGT classes, providing examples for reinforcement and additional explanation. Learners can thus search for newly acquired vocabulary or grammar and see it used in the context of a sentence.

In addition to the search filters, a “Sort” function is also available to organize search results. Users can, for example, sort the videos by shortest or longest sentences first, based on the number of signs per sentence. This function is implemented to reflect a type of sentence complexity where parents of young children might want to prioritize short sentences, while parents of older children or more proficient signers might want to view longer sentences.

#### Viewing videos

When the app user selects a desired sentence, a video featuring a DHH signer signing the sentence is shown. Each video is presented in a loop for continuous viewing.

**Fig. 3:**
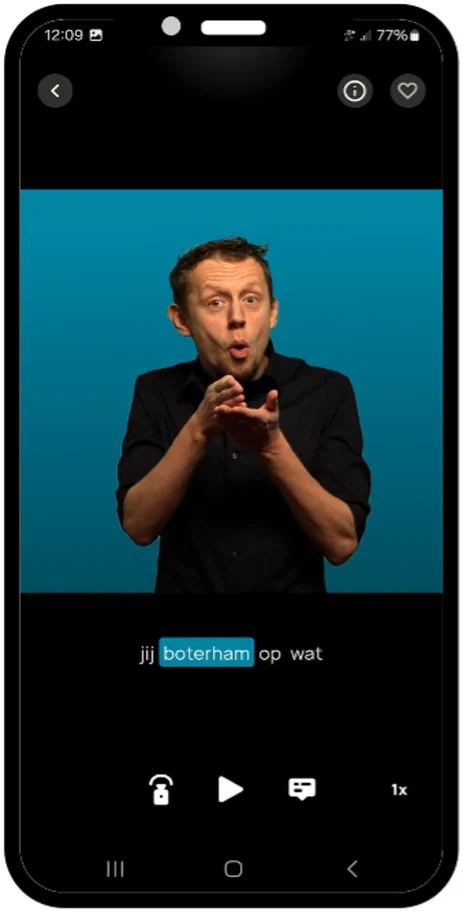
Example video (The signers featured in the prototype videos provided informed consent for the use of their video data within the context of this user study. New videos were recorded before the app was publicly released. For privacy reasons, Fig. 3 shows a newly recorded video rather than the original video used in this user study.)

Underneath the video, as shown in Fig. 3, four different tools can be selected:

- Camera angle: Users can choose between three different camera angles (left, right and center) to view the signer from different perspectives;
- Pause/Play: This tool allows users to pause and resume the video;
- Subtitles: Users can select Dutch subtitles, no subtitles, or a literal sign-for-sign translation (as shown in Fig. 3). In the sign-for-sign translation, the sign currently being produced receives highlighting to aid users in identifying individual signs;
- Playback speed: The video speed can be adjusted between four different settings (0.25 to 1x).

**Fig. 4:**
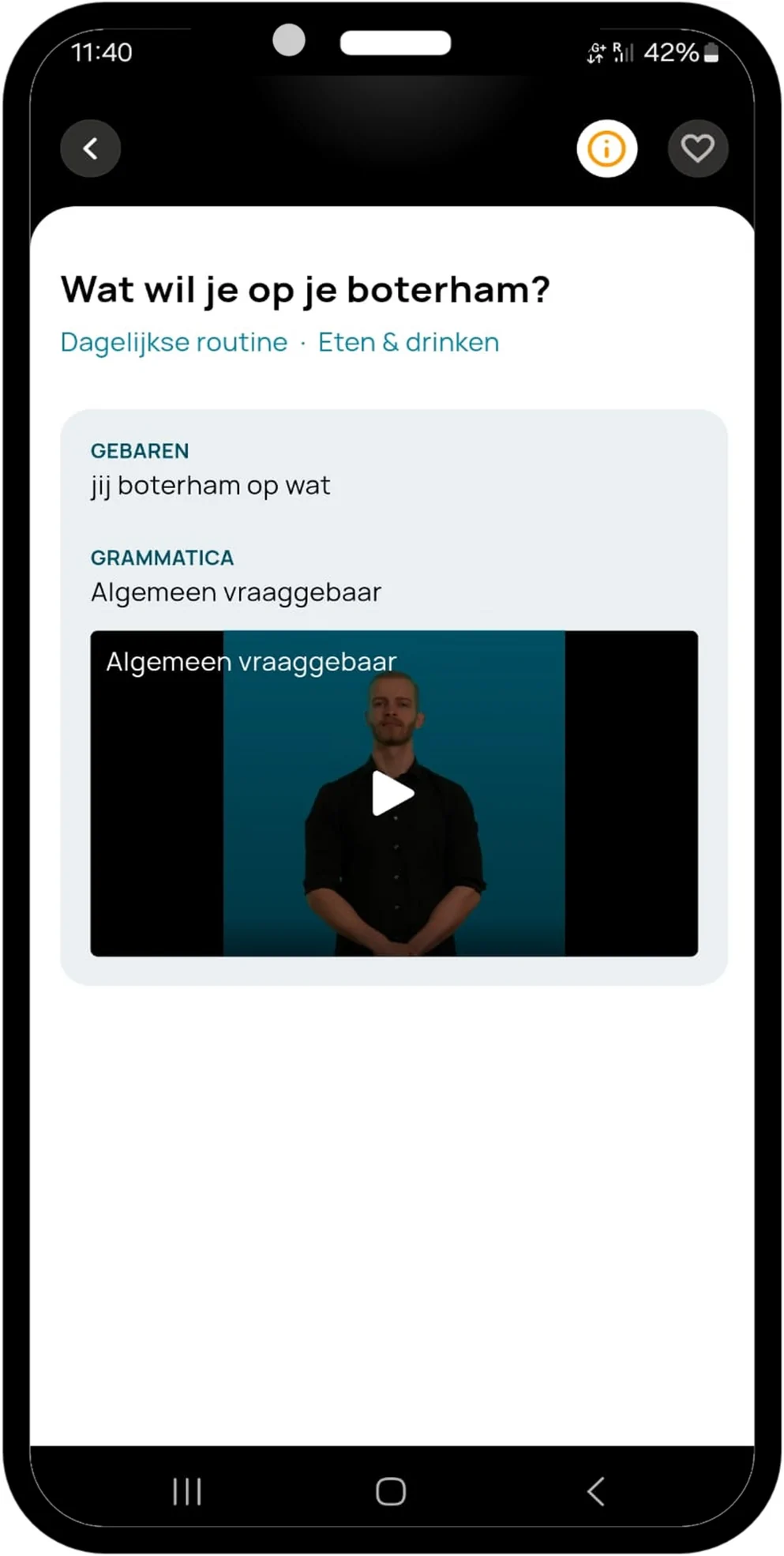
Use of the information button

In the top right corner of the video, two additional tools are available:

- Information: Clicking this tool opens a new window displaying additional information about the video, including relevant grammar explanation videos tied to the sentence (as illustrated in Fig. 4);
- Favorite: Users can mark the video as favorite for easy retrieval.

#### Content

All videos were signed by native DHH signers, who were asked to keep the target group (parents of DHH children aged 1 to 5) in mind to prompt child-directed signing. Their signing was not restricted in any way—for example, they were not requested to use standardized signs or to adhere to basic word order often taught in NGT classes. Although this approach sparked debate among the researchers, it was ultimately decided that the app should reflect the natural variation of NGT as used within the Deaf community.

The current prototype includes 29 example sentence videos to demonstrate the app’s design and functionality. Grammar explanation videos have not yet been integrated; selecting an explanation video currently displays a placeholder.

A note on standardization is important here. Like many other sign languages, NGT is characterized by large regional and social variation, with most of the variation occurring in its basic lexicon [32]. Despite this natural variation, the Dutch government made standardization a requirement for the official recognition of NGT^4^—a decision largely criticized by the Deaf community. This led to a project to standardize the NGT basic lexicon[32].^5^ In educational settings, including parental sign language courses, this standardized lexicon is typically used. This background is particularly relevant in the context of our prototype app, where some signs deviate from these standardized forms.

### Mapping prototype features to design objectives

Having described the main features of the ZINinNGT prototype, we now link these features back to the three design objectives outlined in Sect. 3.1 to clarify the rationale behind the app’s design. As summarized in Table 3, each design objective is supported by specific prototype features.

**Table 3:** Linking objectives to prototype features

Design objective	Prototype feature
Grammar-aware input	Grammar filter Information button Grammar explanation videos
Perceptual control	Adjustable camera angles Adjustable playback speed Subtitle options
Need-driven retrieval	Search bar Search filters Favorites feature

The first objective, grammar-aware input, is supported by: (1) the grammar filter, which allows users to search for sentences containing specific grammatical constructions; (2) the information button, which links NGT sentences to their corresponding grammar explanation videos; and (3) the grammar explanation videos themselves, which offer explanations on NGT grammar and sentence formation.

The second objective, perceptual control, is addressed through the features that allow users to watch videos from multiple camera angles, adjust playback speed, and toggle subtitle options (on or off, sentence- or word-level focused). These features give parents control over how they receive NGT input, accommodating different learning preferences.

The third objective, need-driven retrieval, is supported by the search bar, search filters, and favorite marking option. Together, these features enable parents to efficiently find the videos they need, when they need them, aiming to make the app practical and easy to navigate.

## User study methodology

We conducted a user study with two participant groups: (1) hearing parents of DHH children, and (2) NGT teachers who teach hearing parents. Although the broader project included both semi-structured interviews on parents’ experiences with sign language learning and a user study on the ZINinNGT prototype, this paper focuses only on the latter. For a detailed account of parents’ experiences with sign language learning, see [16]. (Note that part of this work has been discussed in the introduction.)

### Participants

#### Eligibility and recruitment

Hearing parents of DHH children were eligible to participate if they were currently enrolled in or had previously completed an NGT or Sign-supported Dutch (NmG) course. NmG combines spoken Dutch with key-word signing, aiming to make spoken language more visible.^6^ Both NGT and NmG learners were included because, in the Netherlands, hearing parents of DHH children are generally eligible for health insurance-covered parental sign language courses, but in some regions these courses teach NmG rather than NGT. Therefore, including NmG learners allowed us to increase our participant pool and achieve greater geographic diversity. There were no restrictions regarding the age of the participants’ children. Although ZINinNGT is designed for parents of children aged 1 to 5, we wanted to increase our participant pool and believed that parents’ reflections on their past learning experiences could provide valuable insights into what they would have needed in an NGT app.

NGT teachers were eligible to participate if they had experience teaching NGT to hearing parents of DHH children. There were no restrictions regarding the duration of teaching experience or hearing status. It is, however, important to note that the World Federation of the Deaf emphasizes that Deaf people must stand at the forefront of sign language teaching, especially when teaching DHH children and their families [35]. In practice, parental sign language courses in the Netherlands are taught by both DHH and hearing instructors, regardless of whether the course focuses on NGT or NmG. This reflects the fact that the Netherlands has one official teacher training program for NGT (a four-year bachelor’s program^7^), which is open to both hearing and DHH students, with hearing students making up the majority of graduates.

Recruitment took place through social media sites aimed at hearing parents of DHH children, as well as through organizations serving parents of DHH children, such as the Royal Dutch Auris Group, the Dutch Federation of Parents of Deaf Children (FODOK), the mental health and social services for the DHH (GGMD) and Royal Dutch Kentalis, and personal contacts. Participants received a 20-euro gift card upon participation.^8^

#### Parent demographics

A total of 22 parents took part in this study. However, one parent’s data were excluded from the analysis because their child’s age differed substantially from that of the other participants, by 12 years above the mean. This left a final group of 21 parents (17 mothers and 4 fathers), comprising 20 different families. The mother and father from the same family tested the prototype together, while all other participants took part individually.

Our sample was geographically diverse, with participants living in seven provinces across the Netherlands, representing the southern, central, and northwestern regions. Most parents had one DHH child, while one parent had two. The children’s ages ranged from 2 to 9 years, with a mean age of 6 years. All participating parents were hearing, except for one parent who was hard of hearing. Although the study targeted hearing parents, this parent was included because they had no prior experience with sign language before learning that their child was DHH. Finally, parents were asked about their prior experience with educational apps. Thirteen parents indicated that they had used such apps to some extent. A summary of the parent demographics can be found in Table 4.

**Table 4:** Summary of parent demographics (*N* = 21)

	Total
**Gender**	
Female	17
Male	4
**Age of DHH children (years)**	
Mean (SD)	5.57 (2.31)
Median [min, max]	6.00 [2.00 – 9.00]
**Hearing Status**	
Hearing	20
Hard of Hearing	1
**Type of Course(s) Taken**	
NGT	9
NmG	7
NGT & NmG	5
**Prior Experience with Educational Apps**	
Yes	13
No	8

#### Teacher demographics

Six NGT teachers (four female, two male) took part in this study, and all participated individually. Three teachers identified as hearing, and three identified as Deaf. Two of the Deaf teachers considered NGT their first language. The teachers in our sample were rather experienced, with a mean of around 15 years of NGT teaching experience (range: 3–27 years), including teaching groups other than parents. Lastly, all but one teacher reported having used educational apps either currently or in the past. Table 5 summarizes the teacher demographics.

**Table 5:** Summary of teacher demographics (*N* = 6)

	Total
**Gender**	
Female	4
Male	2
**Hearing status**	
Deaf	3
Hearing	3
**First language(s)**	
Dutch	3
Dutch & Frisian	1
Dutch & NGT	1
NGT	1
**Teaching experience (years)**	
Mean (SD)	14.83 (8.23)
Median [min, max]	14.50 [3.00–27.00]
**Prior Experience with Educational Apps**	
Yes	5
No	1

### Testing procedure

This study was carried out by the first author between February and May 2024 and involved a think-aloud procedure. Sessions took place at locations chosen by the participants—typically parents’ homes and teachers’ workplaces. The first author is hearing and a native speaker of Dutch, who learned NGT as an adult and uses it daily in professional contexts. The study was conducted in Dutch, except for two sessions that were conducted in NGT and one in NmG. All sessions were audio-recorded, and NGT sessions were additionally video-recorded.

For the NGT sessions, an NGT-Dutch interpreter was present, chosen according to the participant’s preference. Throughout the study, the researcher and participant communicated directly in NGT with the interpreter providing simultaneous translation into Dutch. This setup allowed the researcher to verify her understanding without interrupting the flow of direct NGT communication. Although the presence of an interpreter influences interactions,^9^ it was considered important to minimize the risk of miscommunication or missing important details in participants’ responses.

Participants were given a smartphone with the ZINinNGT prototype opened on its home screen. They were asked to explore the prototype, click through all available buttons, and share their thoughts and actions throughout the process. The researcher only asked clarification questions during this stage. If the researcher noticed by the end that certain features had been overlooked, the participant was asked to explore those as well.

After testing, follow-up questions were asked, including whether participants would use the app once it became publicly available. Additional questions concerned future additions, such as a voice-over and videos showing conversations in NGT. As these questions were highly suggestive (e.g., “Would you use [feature] if it were implemented?”) and specific to the ongoing development of ZINinNGT rather than to sign language learning apps more generally, they are not included in the analysis presented in this paper.

### Coding and analysis

All sessions were transcribed automatically using Whisper AI software. Transcripts of the Dutch and NmG sessions were manually checked against the original audio recordings, while transcripts of the NGT sessions were verified against the corresponding video recordings. Once verified, all transcripts were imported into ATLAS.ti for coding.

Feedback was coded thematically using four broad categories: *user interface*, *content*, *scope*, and *intended use*. Within each category, additional focused codes (e.g., *video looping*, *native signers*) were applied to capture the specific aspects participants mentioned.

## Results

This section presents feedback on the ZINinNGT prototype from two groups: (1) hearing parents of DHH children learning NGT or Sign-supported Dutch (NmG), and (2) teachers who teach NGT courses for parents. The feedback is organized into four key categories: (1) user interface, (2) content, (3) scope, and (4) intended use—the latter referring to participants’ responses to the question of whether they would use the app once it becomes available.

In this analysis, parents learning NGT and those learning NmG are treated as a single group. This decision was based on the difficulty of reliably distinguishing between the two, as NmG can be considered lying on a continuum between Dutch and NGT. Moreover, many parents attended courses described as NmG blended with elements of NGT. While one might expect that learning NmG versus NGT could influence feedback on an app specifically aimed at NGT learning, making a clear distinction between these subgroups was unfortunately not possible.

### User interface

Feedback on the prototype’s interface was extensive and generally positive. For clarity, the findings are organized into three subsections: (1) search bar and filters, (2) viewing videos, and (3) home screen and app navigation.

#### Search bar and filters

Parents generally appreciated the search tools, particularly the inclusion of the search bar (*N* = 6)^10^ and the semantic theme filter (*N* = 7), which helped them quickly find sentences relevant to their child or daily routines. However, several parents did not understand the purpose of the grammar filter, as it was unclear what selecting this filter resulted in.

Teachers mostly appreciated the favorite filter (*N* = 3) as this would allow parents to save difficult sentences they wanted to practice and easily retrieve them later.

#### Viewing videos

The prototype’s most frequently valued feature among parents was the camera angle tool (*N* = 12), which allowed users to switch between a front, left, and right camera view. Although this feature was new to most parents, many appreciated that it made viewing the signs easier, especially the signs’ handshape—something they often found difficult to see in other materials with only a front camera.

Another highly valued feature was the ability to adjust the playback speed (*N* = 11). Parents mentioned that they often found signing to be very fast, and therefore they appreciated the possibility to slow down the videos without them getting blurry or choppy. The literal sign-for-sign translation with highlighting was also appreciated (*N* = 11), as this provided insight into the sentence structure and sign order. A mother (M) and a father (F) who tested the prototype together explained:

**M: “**And you actually also see the grammatical structure of NGT, because you can read along underneath. That’s actually nice, too. We often do that out loud in class now.”

[…]

**F:** “That’s not the case with the other apps, right?”

**M:** “No.”

**F:** “Then you just get the sentence, and then it’s produced, and then you have to parse it all by yourself.”

[…]

**F:** “But this is really nice. With this, I’m completely… Because when I see *name of teacher* do it, she goes really fast. And then you’re watching the signs, and you’re…”

**M:** “Parsing.”

**F:** “Parsing the sentence. And this is… This really helps a lot.”

Besides the literal sign-for-sign subtitles, parents valued the option to choose between subtitle types (*N* = 8): sign-for-sign translation, sentence-level subtitles or no subtitles. Parents considered being able to switch the subtitles off as helpful, as they viewed this option as a way to test themselves by checking if they could understand the signing without seeing the translation.

Many of the comments made by parents about the user interface were also mentioned by the teachers in our sample. Teachers also mostly valued the different playback speeds (*N* = 5), different camera angles (*N* = 3), literal sign-for-sign translation (*N* = 2) and the general subtitle tool (*N* = 2).

#### Home screen and app navigation

Participants also commented on the overall layout of the app. While parents considered the prototype to have a clear overview, several parents (*N* = 5) and teachers (*N* = 2) found the home screen layout surprising or unintuitive, as they expected to first see a menu (e.g., with tiles or themes), rather than being immediately presented with a list of written sentences.

Several additional usability issues became apparent during testing. Although the information button was intended to link each sentence to its grammar explanation video—an important feature for the grammar-aware input—most participants either overlooked it or assumed it contained general app information, such as privacy policies, and dismissed it.

Two Deaf teachers further commented on the text-heavy design of the prototype, stating that a sign language app should be more visually oriented. They mentioned that because NGT is a visual language, an app should reflect this visual nature. In addition, the teachers noted that some parents come from multilingual backgrounds and may not have strong Dutch reading skills. Including too much Dutch text could therefore make the app less accessible for these parents. As one teacher explains:

“There’s a lot of text in it. […] There’s a strong emphasis on Dutch, and that really demands strong Dutch language skills. And of course, there’s a group of people who aren’t digitally literate. And for them, this app is poor or even not accessible at all. […] It’s not visual, whereas you’d want NGT to be presented as a visual language. And that’s not happening here.”

### Content

Many parents highlighted the app’s focus beyond vocabulary as a major strength, particularly compared to other apps or course materials. Twelve parents appreciated the inclusion of full sentences, and nine specifically valued the grammar explanation videos.^11^ Some parents specifically named the transition from isolated signs to sentences as particularly difficult in learning NGT, and therefore, they appreciated the app as a way to get a feel for NGT grammar and sentence structure. One parent explains about the app extending beyond the vocabulary level:

“We really just missed this, exactly this, because you can’t find this anywhere. […] That’s just because my experience with apps for NGT or NmG or whatever, or courses or… Yeah, it was always just really limited: words, words, words.”

Parents further appreciated that the app content was relevant to young children and their daily lives (*N* = 10). They mentioned that the included sentences were sentences they frequently used with their children, making the content directly applicable in their everyday interactions.

Another topic that frequently emerged during testing was that the signers in the videos often deviated from standardized signs and syntactic structures typically taught in parental courses (*N* = 9). While most parents simply noted that they had learned certain signs differently, some parents saw this as a drawback, expressing a strong preference for the use of standardized signs. During one session where the participant’s child was also present, the child began copying a sign from the prototype, but the parent stopped her, explaining that it was not the correct sign—that is, not the standard sign.

Several parents also drew conclusions about the correctness of signs they had previously learned, adjusting their signing based on the sign variants included in the prototype, assuming they had misremembered the course signs. For others, these differences caused confusion, as they were unsure whether the sign produced in the video or the one learned during their course was the “correct” version.

Teachers’ feedback on the content mostly concerned the correctness and quality of the signing and translations. Five out of the six teachers commented on signs or syntax that they would produce differently during their own lessons. This topic about correctness or standard language use often took up a considerable part of the sessions with teachers. While some did not view these differences as something negative per se, others expressed a clear preference for the use of standardized signs and syntax as used during their own lessons or as described in teacher guidelines:

“Grandmother, oh, again a different sign. grandmother [copies the prototype’s sign for grandmother], I didn’t know that one either. I know grandmother [signs the standardized sign for grandmother], but grandmother [copies the prototype’s sign again], okay. Yeah, no, I would then… Both can be there, but I would also include the standardized one. That helps avoid frustration for parents, I think.”

“[signs] This is how I would sign it grammatically. And the sign for ‘ball’, I would sign it differently. But should that be a concern? Yeah, I don’t know. I follow what’s on the Dutch Sign Center website.^12^ And if parents do homework at home and look this up, and if I then use a different sign than what they might see, that could be confusing.”

Teachers also frequently commented on the correctness of the sign-for-sign translation (*N* = 5). Teachers commented on elements missing in the translation as well as elements they considered incorrect. Most teachers interpreted this translation as a gloss, which led some to comment on the absence of standard glossing conventions—such as the use of small caps.

Positive feedback highlighted that the content was appropriate for children and included child-directed signing (*N* = 2). Teachers noted as an advantage that some sentences reflected language suitable for younger children, while others were more suited to slightly older children. Similar to the parents, teachers also valued that the prototype included full sentences and thus extended beyond vocabulary (*N* = 2).

### Scope of the app

While parents appreciated that the prototype went beyond vocabulary, many parents expressed surprise that it did not include separate vocabulary at all (*N* = 8). While some parents only expressed surprise at not seeing vocabulary, others considered this a disadvantage, as it made the prototype less holistic. They felt they would need to use an additional app or tool to look up individual signs. One parent explained:

“The only thing is, you think, yeah, you’re in this app because you want to look something up. And then you don’t want… *Shall we make a nice card for grandma?*, it says here. And then, of course, more content will be added. But then if you want to say *Shall we make a nice card for Uncle Piet?*, then you think, oh shoot, what’s the sign for *uncle*? Yeah, that’s something you can’t quickly look up then.”

Five parents, therefore, suggested adding vocabulary to the app. In addition, six parents proposed including quizzes to test and challenge themselves. Several also mentioned appreciating how some other materials make signs’ handshapes more explicit—for example, through an icon next to the video depicting the correct handshape or by a short demonstration of the handshape before producing the sign itself. Because handshapes are often difficult to see, parents expressed a wish to include such features in the app as well.

Beyond quizzes, some parents expressed that they missed a broader form of gamification (*N* = 5), such as the ability to compete against other users, the addition of motivational elements, or the addition of fun games within the app.

Similar to the parents, teachers expressed a desire for separate vocabulary, quizzes, and explicit handshape information (each mentioned in two interviews). They explained that quizzes would help parents practice and reinforce what they had learned. Information on handshapes was also valued, as handshapes are often challenging for parents and usually receive extra attention in class. Teachers also emphasized the importance of making the app more accessible to parents with different language backgrounds who are not proficient in written Dutch, suggesting the inclusion of translations into other languages such as Arabic or Turkish (*N* = 2). Finally, two teachers indicated that they would like the subtitles to be interactive, allowing parents to click on individual words to view videos of isolated signs, related vocabulary, or sign variants.

### User intent

Parents with children within the target age group (1–5 years) were asked whether they would use ZINinNGT if it became available with the same design but more content. Parents with older children were asked whether they would have used the app when their child was still younger. Teachers were asked whether they considered the app useful for parents.

Eighteen of the twenty-one parents indicated that they would use ZINinNGT, citing the following main reasons:

- ZINinNGT fills a gap in current sign language learning materials by offering more than just vocabulary;
- It is easily accessible on a mobile phone and does not require logging in;
- The content is highly relevant to parents with young children;
- There is a general lack of available sign language learning materials, so any new resource is very welcome.

Parents mentioned various moments and goals for which they would use the app. Some said they would use it as an on-the-spot translation tool during daily interactions with their child. Others indicated they would use it to prepare for specific situations, for example, by going through the theme “animals” before visiting the zoo with their child. Most parents, however, mentioned wanting to use the app as a learning tool during spare moments. Although the app was not designed to be attractive to children, some parents also said they would like to use it together with their child as a shared activity or for joint learning, indicating a large wish for material that can be used together by parent and child.

Among teachers, four of six considered the app useful for parents. One teacher felt unable to judge its usefulness but would inform parents once it became available, while another noted it would be useful provided it also included separate vocabulary. Teachers’ reasons largely aligned with those of parents: they valued the inclusion of full sentences and grammar explanation, as well as the app’s practical and parent-focused nature, and they also mentioned the general lack of NGT learning materials. One teacher emphasized that most parents are eager to make use of any available sign language material.

## Discussion

This study explored feedback from hearing parents of DHH children and NGT teachers on the ZINinNGT prototype—an NGT learning app for parents focused on sentence formation. While participants provided detailed feedback on specific features, several broader themes emerged that can inform both the future development of ZINinNGT and other sign language learning tools.

### Validation and extension of design principles

In this section, we discuss the original design principles presented in Sect. 3.1 and extend them based on insights gained from our user study.

First, our findings reinforce the importance of going **beyond the vocabulary level** in sign language materials. Both parents and teachers viewed the app’s focus on full sentences and grammar explanation as a key strength. This aligns with earlier research showing that hearing parents wish for more instruction on sentence structure in sign language courses and resources [14–16]. The use of a literal sign-for-sign translation, with highlighting of the currently produced sign, was seen as effective in making sentence structure more transparent. These findings suggest that future tools should focus on supporting broader language development rather than focusing solely on vocabulary learning.

Second, several features that enabled **perceptual control** were viewed very positively. Participants appreciated the adjustable playback speed, multiple camera angles, and different subtitle options, all of which were seen as helpful for learning.

Third, the possibility to use the search bar, mark videos as favorites, and filter by semantic theme were considered helpful in content retrieval. More broadly, this highlights the principle of **need-driven retrieval**: parents valued being able to quickly find the content most relevant to their learning objectives or daily communicative needs.

Fourth, many parents expressed a desire for a more **holistic** app—one that includes sentences and grammar explanation, but also lexicon and quizzes. They preferred a single platform where they could study grammar, look up signs and practice what they had learned. This also underscores the importance of interactive activities, such as quizzes, aligning with the Multiple pedagogies/activities principle described by Jamaldeen, Hewagamage, and Ekanayaka [29] and with other previous literature [30].

Fifth, parents missed design features related to stimulating **learner motivation**. They expressed a wish for gamification elements, again aligning with the guidelines of Jamaldeen, Hewagamage, and Ekanayaka [29] and other research that emphasizes the importance of implementing gamification to increase learning motivation and perseverance [30, 31].

Sixth, teachers emphasized the need for a **visual-first** approach. A sign language app for parents should keep text to a minimum, matching the visual nature of sign language and making it more accessible for parents from different language backgrounds.

A seventh key theme was the tension between standardization and natural variation in NGT. While the app intentionally aimed to reflect sign language as used within the Deaf community, some participants expressed confusion when signs or grammar differed from what they had previously learned. This raises a broader question for sign language learning tools: should they prioritize standardized forms for consistency, or embrace variation to reflect natural language use? A possible middle ground is to explicitly **acknowledge variation**—for example, by including sign variants and an explanation of natural variation—so that learners gain awareness without becoming confused. It is also important to note that sentence-level signing involves more variation than individual signs, and there is no one-to-one correspondence between written sentences and their signed equivalents. The goal of a sentence-focused tool should therefore not be for parents to memorize full sentences, but to develop the ability to form their own, which can be difficult to achieve without teacher guidance. Language learning apps should be viewed as a supplement to language courses rather than a replacement thereof. As found by Jamaldeen, Hewagamage, and Ekanayaka [29], mobile language learning tools are less effective when used as a primary means of learning rather than being integrated in a broader learning environment.

Lastly, several parents mentioned that they imagined using ZINinNGT together with their child. While ZINinNGT was not designed for young children (it lacks fun images, colors, and games), this feedback reveals a clear interest in material that can be used together by parent and child. This aligns with findings reported in our previous publication [16], in which we found that parents and teachers value self-study resources that can be used for **joint parent–child learning**. Future tools may benefit from incorporating elements attractive to children to support joint sign language learning between hearing parents and their DHH children.

In summary, based on the results of our user study, we validated our original three design principles and extended them to the following eight:

1. Grammar-aware input;
2. Perceptual control;
3. Need-driven retrieval;
4. Holistic design;
5. Design for learner motivation;
6. Visual-first;
7. Variant-aware;
8. Joint parent–child use.

### Limitations of the study

This section outlines several limitations of our study that may have influenced the findings and their generalizability.

First of all, the study focuses on design principles derived from a short-term user study. While design features can contribute to a productive and motivating learning environment, app design by itself does not ensure good learning outcomes [31]. With this study, we aimed to provide insight into the design principles required for sign language learning apps. However, the study does not provide insight into users’ learning trajectories nor the aspects that contribute most to learning gains (see [31] for suggestions for potential research questions when evaluating gamified language learning apps). Therefore, to gain a better understanding of the effectiveness of a language learning app, it would be largely beneficial to include second language learning frameworks and conduct a long-term evaluation study focusing on both learning experiences and objective learning outcomes. For example, this could involve conducting quantitative pre- and post-tests on various language skills, combined with user experience surveys or interviews. We plan to conduct such a study once ZINinNGT is ready for public use.

Second, our recruitment call was written in Dutch, and all interviews with parents were also conducted in Dutch. This, of course, implies that participants were fluent in the language. However, in the Netherlands, 12% of DHH individuals are first-generation immigrants and 20% are second-generation immigrants, reflecting large ethnic and linguistic diversity [39]. Teachers in our sample also mentioned large linguistic diversity amongst parents in their courses and therefore suggested making ZINinNGT less text-heavy and adding translations into other languages. Our parent participants did not raise language accessibility issues—likely because they did not reflect the diversity present in the broader population of parents of DHH children in the Netherlands.

Further, when asking participants about user intent, there is a risk that participants will respond positively to all new materials simply due to a general lack of sign language resources. Both parents and teachers expressed appreciation for new materials being developed, making it difficult to determine whether their enthusiasm was specific to our app or more broadly directed toward any sign language learning tool. Additionally, participants were aware that they took part in a study focused on a sign language app, which likely made them more open to the idea of an app—possibly more so than the average parent or teacher. Still, their explanations for potential use provide meaningful insights into the features they appreciated and the contexts in which they imagined using the app.

Finally, observing participants as they tried out the prototype is not representative of real-world app use. The presence of a researcher likely influenced participants’ behavior, and sharing their thoughts aloud may have felt awkward or unnatural to some, potentially affecting the authenticity of their responses.

### Further development of the app

Based on the feedback from parents and teachers, we have been working on a new version of ZINinNGT that addresses the issues and wishes identified in our study. To create this new version, we collaborated with a Deaf-led company^13^ that focuses on teaching and spreading information about NGT and Deaf culture. A first beta version of the app—now called Signio—was released in September 2025.^14^ Signio is aimed at a broader target audience but will include a dedicated section for parents that is free to use and features example sentences and grammar explanation videos. At the time of writing, Signio has approximately 13,100 registered users. It is also possible to use the app without an account (but we do not have data on the number of unregistered users).

**Fig. 5:**
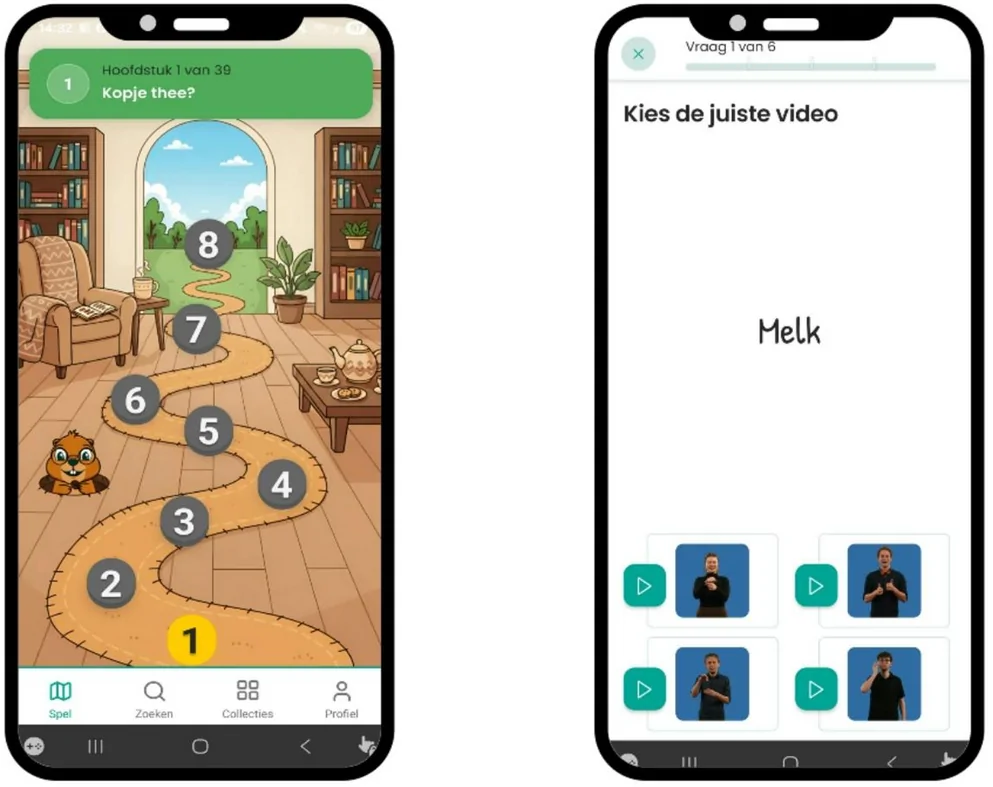
Screenshots of the new version of ZINinNGT showing gamification and a quiz question

To create a more holistic learning tool (additional design principle 4), we expanded the app by including separate vocabulary, quizzes, and gamification elements. The quizzes currently focus on vocabulary comprehension, while the gamification features allow users to earn points, complete levels and track their progress, making learning more motivating (additional design principle 5), see Fig. 5.

To make the app more visual—and less textual—(principle 6), we redesigned the interface to include additional icons, increased use of color, reduced text, and a visual-first home screen.

In line with principle 7 (variant-aware design), we included both standardized and regional variants in the new vocabulary section and plan to add explanation videos on natural variation in NGT. By doing so, we aim to reduce parents’ confusion about the “correctness” of signs and increase their awareness of natural linguistic variation.

At the time of writing, the app includes 3,349 videos of example sentences specifically created for hearing parents of DHH children. Future plans include expanding the app to 4,300 videos of example sentences (all of which have already been recorded and subtitled), adding approximately 30–40 grammar explanation videos with topics particularly relevant for parents (such as getting attention, child-directed signing and use of conditionals) as well as videos on Deaf culture.

**Fig. 6:**
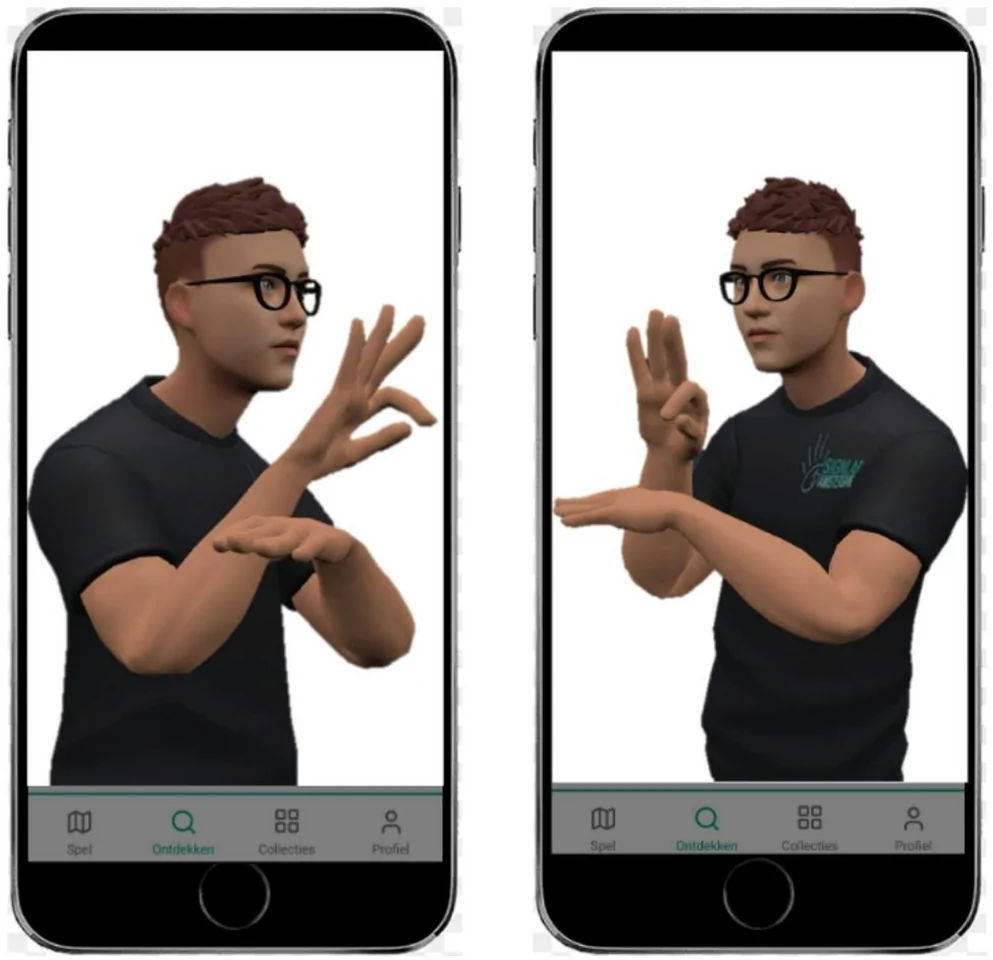
Screenshots of the new version of ZINinNGT showing an avatar from different angles

In addition, all sentences have also been recorded as 3D animations using motion capture technology, as we aim to use signing avatars to generate up to 10,000 sentence videos by making use of compositionality. This means that, by combining words or phrases, we aim to create new sentences from existing elements (for example, combining “Do you want an apple?” and “There’s a pear on the plate.” to generate “Do you want a pear?” and “There’s an apple on the plate.”) The use of 3D avatars also allows for a 360-degree view of the signing, as well as a zoom function to clearly distinguish different handshapes (see Fig. 6)—a solution to the difficulty of adding handshape information to full sentences.

To further enhance engagement and support joint parent–child learning, we plan to incorporate videos of stories in NGT signed by both human and virtual signers, including cartoonish or non-human characters. For children, it is especially important to create an engaging and interactive learning environment [42]. Moreover, research indicates that the use of colorful cartoon characters can stimulate children’s curiosity, thereby enhancing engagement and facilitating language learning [30]. By visual enhancements and by integrating quizzes, a structured learning path, NGT stories, and cartoon characters, we aim to make the app appealing to children and support joint parent–child learning (design principle 8).

Lastly, Jamaldeen, Hewagamage, and Ekanayaka [29] identified the incorporation of collaborative activities as a core facet of mobile learning design. Wiboolyasarin and Jinowat [30] also identified collaboration—besides personalization, interactivity, and gamification—as one of the design features that are most effective in enhancing the language proficiency of primary school children. In the Signio app, different thematic collections are integrated, and users can create and share their own collections. The ability to create personalized collections contributes to the need-driven retrieval and personalization of the app, while sharing collections may be seen as a first step towards collaboration. Although not identified as a separate design principle in this study, adding actual collaborative activities to the app may be beneficial—especially when aiming to make Signio attractive to children.

## Conclusion

Feedback on the ZINinNGT prototype showed that both parents and teachers appreciated its focus on sentence formation, perceptual control, need-driven retrieval, and parent-focused content. Based on feedback from our user study, we also identified additional design principles for future developments and sign language learning tools in general: a more holistic design, design for learner motivation, visual-first approaches, variant awareness, and joint parent–child use. A sign language app can be a valuable complement to sign language courses by providing extra content and practice, but it is not a replacement. Opportunities for real-life interaction with teachers and Deaf signers remain essential for interactive learning, cultural exposure, and personalized feedback. Still, we believe that tools like ZINinNGT can support hearing parents of DHH children in their daily learning process, particularly when course access is limited.

Finally, our findings confirm the broader need among hearing parents for more sign language learning resources. We hope that ZINinNGT can contribute to filling this gap. By developing learning tools specifically tailored to the needs of parents, we can support them in learning sign language and, in doing so, help improve DHH children’s access to language.

## Data Availability

The data generated by this study are not openly available due to privacy considerations. Because the community is very small, it is unfortunately not possible to fully prevent personal identification if the data were to be shared.

## Footnotes

1 The name ZINinNGT is a word play, meaning both “Sentence in NGT” and “In the mood for NGT” in Dutch.

2 These are the *KinderGebaren-app* [18] and the *Lotte en Max Kindergebaren app* [19].

3 In this paper, ‘deaf’ with a lowercase d refers solely to audiological status, while ‘Deaf’ with an uppercase D refers to the cultural and linguistic minority group that uses sign language.

4 In 2021, NGT was officially recognized.

5 This project is called Standardization of Basic Lexicon (STABOL). Standardization was based on regional distribution, frequency, formality, clarity and educational use, see [32].

6 However, in practice, NmG often fails to represent either Dutch or NGT clearly, making it difficult to understand for those who rely primarily on visual input [33, 34].

7 See Inhoud & vakken Leraar Nederlandse Gebarentaal/Tolk NGT—Voltijd | Hogeschool Utrecht for more information on the teacher training program NGT [36].

8 This excludes three teachers who were contractually required to participate in scientific research.

9 See for example [37] on interpreter influences.

10 Throughout this paper, *N* refers to the number of sessions in which a comment was made, not the number of individual participants (as one session involved two parents). For the parents, total *N* = 20, and for teachers, *N* = 6.

11 Note that grammar explanation videos were not included in the prototype yet; parents could only see placeholder videos and some of the potential topics.

12 The Dutch Sign Center website refers to the online NGT dictionary [38], which includes the standardized signs as described in Sect. 3.2.3.

13 See TYD | Nederlandse Gebarentaal voor iedereen [40].

14 For more information and download see Signio-app—Leer Nederlandse Gebarentaal [41].
